# Diabetic Ketoacidosis: Possible Cause of Thrombotic Thrombocytopenic Purpura

**DOI:** 10.7759/cureus.18017

**Published:** 2021-09-16

**Authors:** Logan J Jackson, Hannah Fischer, Nardine Abdelsayed, Mary Carter

**Affiliations:** 1 Internal Medicine, Grand Strand Medical Center, Myrtle Beach, USA; 2 Internal Medicine, Edward Via College of Osteopathic Medicine-Carolinas, Myrtle Beach, USA

**Keywords:** diabetic ketoacidosis, thrombotic thrombocytopenic purpura, adamts13, microangiopathy, plasma exchange, microscopic hemolytic anemia, coagulopathy

## Abstract

Acquired thrombotic thrombocytopenic purpura (TTP) is an uncommon microangiopathic disorder that can have variable presentations and can be precipitated by a multitude of stressors to the body, most commonly sepsis. TTP is caused by a deficiency of ADAMTS13 leading to intravascular clotting causing thrombocytopenia and microangiopathic hemolytic anemia. TTP can be associated with various other pathologic conditions. One such rare association has been reported with diabetic ketoacidosis (DKA). Here, we present an even less appreciated presentation in association with DKA. A 62-year-old African American male with previously diagnosed prediabetes presented with DKA and developed hemodynamically significant bleeding. He was confirmed to have TTP that responded to plasmapheresis. TTP is a life-threatening illness if not treated urgently with plasmapheresis with or without rituximab. As acquired TTP most commonly occurs during stress on the body, it is important to treat the underlying stressor. Early identification and initiation of appropriate interventions are crucial to reducing mortality associated with TTP. Furthermore, we need to appreciate less commonly associated conditions such as DKA among patients.

## Introduction

Thrombotic thrombocytopenic purpura (TTP) is a microangiopathic disorder in which the blood coagulates in vasculature and organs, rapidly depleting platelets and causing end-organ damage most commonly affecting the brain, heart, and kidneys [1-3]. TTP is a specific disease under the subcategory of thrombotic microangiopathies classified by a severe deficiency of the protein ADAMTS13 (which is a disintegrin and metalloproteinase), which cleaves von Willebrand factor (VWF) multimers. This allows uncleaved VWF multimers to accumulate and recruit platelets for massive intravascular clotting [1,4]. TTP has a unique pentad of characteristics: thrombocytopenia, renal failure, microangiopathic hemolytic anemia, fever, and neurological deficits. However, not all patients experience each symptom simultaneously. Due to the high mortality associated with TTP, it must be suspected in the setting of thrombocytopenia occurring concurrently with hemolytic anemia so that prompt treatment with plasmapheresis can be initiated [1,5,6]. TTP is classically triggered by sepsis, toxins, autoimmune disease, radiation, transplant, malignancy, pancreatitis, or cardiovascular surgery, and is reported in a few cases of diabetic ketoacidosis (DKA) [1,3,5-7]. Here, we report the development of TTP in a man who presented with new-onset diabetes and DKA.

## Case presentation

A 62-year-old African American male with a medical history of prediabetes presented via emergency medical services (EMS) to a level one trauma center for altered mental status and hyperglycemia. He reported mild generalized abdominal pain, thirst, and fatigue for several days before arrival. Additionally, his wife noticed acute onset of confusion and abnormal behavior which prompted her to call EMS. Physical examination was unremarkable except for disorientation to time and tachycardia to 115. He had no signs of bleeding. Initial labs revealed hyperglycemia of 670 mg/dL, anion gap of 20 mEq/L, bicarbonate of 16 mmol/L, pH of 7.192, potassium of 4.7 mmol/L, and ketonuria with HgbA1c of 10.7%. The patient was also anemic (9.1 g/dL) and severely thrombocytopenic (10,000/mm^3^) with elevated blood urea nitrogen (78 mg/dL) and creatinine (2.4 mg/dL), consistent with an acute kidney injury. Total bilirubin was elevated (2.9 mg/dL) as well as aspartate transaminase (59 U/L); however, other liver function tests were within normal limits. Given the laboratory findings, the patient was started on an insulin drip, intravenous (IV) fluids, was typed and screened, and platelets were ordered. After transfusion of one unit of platelets, repeat labs showed an increase to 46,000/mm^3^. Coagulation studies showed the international normalized ratio of 1.32, prothrombin time of 15.1 seconds, and partial thromboplastin time of 25.9 seconds. Furthermore, fibrinogen was elevated at 484 mg/dL, haptoglobin was low at <20 mg/dL, lactate dehydrogenase (LDH) was >2,800 U/L, reticulocyte count was 5.5%, and peripheral smear was positive for schistocytes. Several hours later, his mental status declined and he became unresponsive to any questioning. However, he was able to move all extremities but not on command, and his repeat platelet count was 10,000/mm^3^.

Based on the symptoms and concurrent laboratory abnormalities, there was a concern for microangiopathic hemolytic anemia in addition to DKA. Because the patient did not take any medications, the differential diagnosis included disseminated intravascular coagulation, TTP, sepsis, and hemolytic uremic syndrome. The patient’s severe thrombocytopenia with schistocytes on peripheral blood smear made TTP the most likely diagnosis. Therefore, a tertiary care center was contacted for the transfer of the patient as plasmapheresis could not be done in-house. ADAMTS13 was tested, and methylprednisolone (90 mg IV) and four units of fresh frozen plasma were administered to bridge the patient through transport. The patient decompensated upon arrival of the transport team as he became acutely hypoxic requiring intubation, during which blood was noted in the oropharynx. Repeat hemoglobin at this time was 5 g/dL. Hemodynamic stability was achieved with two units of packed red blood cells (PRBCs) and the patient was sent to another facility. During transfer, the patient became acutely hypoxic and hypotensive. Upon arrival to the facility, he had bright red blood oozing from the oropharynx and bilateral nares. The patient was immediately given two additional units of PRBCs and one unit of platelets. At this time, the cardiac index was 1.9 L/minute/m^2^ with a systemic vascular resistance of 1,492 mmHg×minute/mL. Norepinephrine drip was initiated and titrated to maintain organ perfusion. A bedside transthoracic echocardiogram demonstrated global hypokinesis with underfilled ventricles bilaterally, along with moderate pericardial effusion without tamponade physiology. On repeat physical examination, the patient was tachycardic with pinpoint pupils, distant heart sounds, diffuse petechiae throughout, and displayed purposeful movement of all four extremities. Repeat platelet count was 18,000/mm^3^, and hemoglobin was 7.5 g/dL. The patient’s PLASMIC score for TTP was calculated at 7, indicating there was a 72% chance he was suffering from TTP. Empiric therapy was initiated with plasmapheresis twice daily until his LDH was approximately 500 U/L (1,552 U/L at presentation). The patient was also treated with methylprednisolone and rituximab after plasmapheresis. ADAMTS13 eventually reached an undetectable level, thus confirming the presumed diagnosis of TTP. The neurologic function returned to baseline 24 hours after initiation of plasmapheresis, and renal function recovered before discharge. After a 10-day hospital course, he was discharged home following a return to his baseline functioning. The patient was prescribed insulin at discharge. He was scheduled to follow up with his primary care provider for further workup of his diabetes given his presentation of DKA with only a prior diagnosis or prediabetes. In addition to primary care follow-up for diabetes, he will continue to follow with hematology due to the lifelong risk of relapse of TTP.

## Discussion

TTP is an autoimmune phenomenon that includes both hereditary and acquired forms (aTTP), with acquired being the most common. aTTP has an estimated prevalence of two cases per million per year, with seven times higher incidence among African Americans compared to other ethnicities. The median age of onset is in the fourth decade of life, with a wide range between the third and sixth decade [1,8].

TTP can present as a primary disease or can be provoked by a body stressor, including autoimmune disease, infection, malignancy, and pregnancy [1]. aTTP is classically triggered by sepsis, toxins, and autoimmune disease among others, but not much is known about the association between DKA and aTTP [1,3,5-7]. DKA can commonly present with electrolyte abnormalities, shock, cerebral edema, and other correctable abnormalities. More serious and less common complications include arrhythmias and acute kidney injury [9]. Although coagulopathy in the setting of DKA is rarely reported, few cases have been reported [3,5,6,10]. Even though there is no concrete association between TTP and DKA, a relationship between the acute presentation of DKA with TTP has been postulated. However, the extent to which the two are connected remains unclear. Our interpretation is that one of the presenting acute illnesses precipitated the other, with DKA causing the presentation of TTP to be more likely of the two. aTTP has several documented triggering conditions in which the body is stressed acutely or chronically, including infection, organ transplant, and pregnancy. It is possible that acute stress in the setting of DKA can precipitate TTP through mechanisms that are not currently understood.

TTP requires urgent treatment to prevent death. To determine a patient’s risk of TTP, in case of a PLASMIC score greater than 5, a presumptive diagnosis can be made and treatment can be started. The current gold standard of treatment is plasmapheresis [11]. Since the use of plasmapheresis has begun, the mortality rate has dropped from 90% to 10-20% [11]. Glucocorticoids should also be administered along with plasmapheresis. The benefit lies in decreasing the production of ADAMTS13 autoantibodies, as well as in decreasing cytokine production and lowering the clearance of ADAMTS13 through autoantibodies. In addition to plasmapheresis and glucocorticoids, patients should also receive rituximab at initial presentation to prevent relapse [12].

Once patients develop TTP, they will need to be regularly followed to prevent and treat relapses. Most relapses occur within the first week after stopping infusions, and thus strict lab work needs to be followed, specifically complete blood count to monitor thrombocytopenia or anemia. Patients initially treated with glucocorticoids and plasmapheresis without the use of rituximab have an approximately 50% chance of relapse, highlighting the use of rituximab on initial presentation [13]. Continuous monitoring of patients is recommended even after the initial diagnosis. Relapses can occur years after the first diagnosis with nonspecific symptoms such as anemia, headache, gastrointestinal symptoms, or focal neurologic changes. Therefore, patients with a history of TTP and any acute illness need to have platelet levels checked regularly. If the platelet count is below 100,000/µL the patient should be hospitalized.

There are associations between TTP and the development of other chronic illnesses such as other autoimmune diseases including lupus, depression, renal failure, and neurocognitive dysfunction [14]. Therefore, follow-up with these patients is recommended to assess for any other complications that arise as a result of TTP.

Because aTTP is typically associated with underlying acute illnesses, part of the treatment plan needs to include treating these different pathologies. Our patient presented with DKA and was simultaneously treated for his acute TTP as well as DKA. It is important to treat the underlying condition while administering plasmapheresis to help prevent relapse of TTP.

## Conclusions

To investigate a correlation between the two disease states, patients presenting with DKA and thrombocytopenia should be carefully monitored. Key symptoms to monitor patients with DKA include thrombocytopenia, fever, neurologic symptoms, renal injury, gastrointestinal symptoms, elevated LDH, and reduced hematocrit. Unfortunately, most symptoms of aTTP and DKA are similar. Therefore, we suggest that for patients with DKA and anemia or thrombocytopenia, a peripheral blood smear should be performed to look for schistocytes. In addition, these patients would benefit from haptoglobin and LDH testing to assess for hemolytic anemia. Further investigation of the relationship between TTP and DKA is warranted to identify the possible causality between the two critical illnesses. Clinicians must be wary of thrombocytopenic diabetic patients presenting acutely as to not delay life-saving treatment.
